# Transformative truths: discovering how social media influences the adultification of Black girls through a youth participatory action research (YPAR) approach

**DOI:** 10.3389/fpsyg.2026.1844718

**Published:** 2026-09-28

**Authors:** Meagan N. Scott, Jennifer L. Doyle, Maya K. Knight, Judith Owusuaa-Foster

**Affiliations:** University of Kentucky, College of Education, Lexington, KY, United States

**Keywords:** adultification, Black girls, mental health, social media, YPAR

## Abstract

This study explored how social media influences the adultification and sexualization of Black adolescent girls in a Southern city located in the United States. Using a Youth Participatory Action Research (YPAR) framework and photovoice methodology, the study centers the perspectives and experiences of Black adolescent girls. Five Black high school girls participated as youth researchers and learned all stages of research through a series of structured workshops. Through photovoice, they learned how to capture and analyze photos, captions, and descriptions reflecting their lived experiences with social media as Black girls. Guided by a three-step analysis process and a secondary analysis, five themes emerged: (1) the juxtaposition of childhood innocence and adultification, (2) the lack of role models, (3) the impact of others’ voices, actions, and opinions, (4) Western beauty standards: us versus them, and (5) the overlooked impact on mental health. Findings highlighted how social media functions as a powerful tool that produces, reinforces, and internalizes harmful narratives about Black girlhood. At the same time, participants demonstrated critical awareness and resistance to these messages. Implications underscore the need for school and community-based interventions, including culturally responsive media literacy, educator training on adultification bias, and the integration of youth voices into decision-making processes. This study contributes to the literature by centering the perspectives of Black girls and illustrating the utility of photovoice within a YPAR framework. Future research should expand upon these findings by exploring longitudinal impacts and evaluating culturally-informed interventions designed to disrupt harmful social media influences and promote positive developmental outcomes.

## 1 Introduction

Black youth in the United States continue to navigate systems and institutions historically structured by racial inequity and oppression (Finigan-Carr and Sharpe, 2024). Because of their lived experiences, Black students hold intimate knowledge about institutional racism and other forms of oppression that they face during their everyday encounters (Bell, 1992). More specifically, Black girls, due to their intersecting identities of race, gender, and age, are often impacted by systems of oppression in their community. Eurocentric, heterosexual, ableist, middle-class values and expectations are often placed on Black girls, and as a result, they face constant discriminatory policing of their behaviors, appearance, and social-emotional regulation abilities (Blake et al., 2017; Simmons, 2021). The policing of Black girls is not a new concept, as Black girls have been viewed as less innocent and feminine than their white^1^ counterparts, which is known as adultification (Epstein et al., 2017). However, because Black girls endure negative, spirit-harming experiences, combined with the stressors of gendered racial trauma, they are a vulnerable population who are at risk for more externalizing and internalizing behavioral, relational, and mental concerns in their communities (Sheftall et al., 2022). However, Black girls’ communities can also be a source of joy and opportunity. For instance, Black girls can be involved in communities that uplift and support the strengths that they bring to the table. These spaces can also optimize the usage of counter-narratives of Black girls, thus resisting the predetermined narrative that society places on Black girls (Solórzano and Yosso, 2001).

Currently, there is a need for more literature that centers Black girls and their social, emotional, and behavioral outcomes at sites of oppression and opportunities within their communities (e.g., physical and online spaces) with care and attention, especially since Black girls’ experiences are often silenced when compared to Black males (Evans-Winters and Esposito, 2010; Hood, 2023). In educational spaces, Black girls’ experiences are often aggregated with Black males’ experiences in order to explore research related to Black youth as a whole, and research rarely focuses on the intersection of race and gender (Blake et al., 2011; Young et al., 2021). Furthermore, when or if Black youth experiences are discussed in educational and youth-focused research, the scholarship often perpetuates negative narratives of deficiency (Tuck, 2009), pathology (Ferguson, 2000), or white saviorism (Baldridge, 2014; Matias et al., 2020). Agger et al. (2024) also noted in a systematic review that the intersecting social identities of Black girls in adolescent-based research are often not acknowledged and that the field heavily focuses on quantitative data. Therefore, to better understand the experiences of Black girls within their local communities and systems of oppression and opportunity that operate within them, it is essential to include the voices of Black girls by developing an authentic partnership, which can be done through participatory methodologies. Black girls are experts in their experiences and, when given the opportunity, are capable of addressing problems that affect their well-being (Ozer, 2016).

This paper outlines a qualitative Youth Participatory Action Research (YPAR) project that uses a photovoice methodology with five Black, female adolescents (youth researchers^2^) from the South. With the adult researchers facilitating the conversation, the youth researchers exclusively developed the research topic and question, choosing to study the following area: *How does social media influence the adultification and sexualization of teenage Black girls in their local community?* By co-researching with the students rather than conducting research on them, this paper argues that (1) Black girls produce rigorous, community-rooted knowledge and (2) that social media is not always a neutral space for Black girls, and instead of it being a place of joy, it can often feel like a site of oppression where adultification, colorism, beauty standards, and the erasure of Black girlhood are actively reinforced. Social media is already a prevalent and pervasive reality of adolescence (Anam and Harness, 2025; Homayoun, 2018). Thus, this paper argues that adult researchers, school practitioners, caregivers, and community members should draw on the lived experiential knowledge and wisdom of those most impacted to drive meaningful change in communities that shape the daily functioning of Black girls.

## 2 Literature review

### 2.1 Current experiences of Black youth

Black youth are impacted by intersecting systems of oppression that stem from structural and symbolic forms of violence (Finigan-Carr and Sharpe, 2024). In recent years, more videos showing police violence in the United States have made it more visible how much inequality and discrimination Black communities continue to face (Richardson and Ragland, 2018). Academically, decades of research show that Black students are disproportionately subjected to exclusionary discipline practices, including suspensions, expulsions, and referrals to law enforcement, often for behaviors that are interpreted as less severe when displayed by their white peers (Skiba et al., 2014). Recent national data support these findings. Data from the United States’ Centers for Disease Control and Prevention [U.S. CDC]’s 2023 Youth Risk Behavior Survey highlighted how often high school students reported being disciplined unfairly and how this related to their school experiences, mental health, and risky behaviors (2023). About one in five students reported being treated unfairly at school in the past year, with Black students reporting this more often than white and Hispanic students (United States Center for Disease Control and Prevention, 2023). However, amongst Black youth, Black girls are a historically invisible population whose experiences are often devalued and dehumanized, and mainstream discourse about gender and race often focuses mainly on perpetuating society’s narratives about Black girls, thus normalizing the exclusion of their realities and lived experiences in their communities (Morris and Perry, 2017).

#### 2.1.1 Current experiences of Black girls

To understand and conceptualize the unique experiences of Black girls, the Black feminist thought theory provides a framework that emphasizes cultural perspectives and ways of knowing, while also recognizing the interconnected effects of culture, race, class, gender, and historical marginalization on the lived experiences of Black women and girls (Hill Collins, 2000; Lindsay-Dennis, 2015). Unlike traditional theories that often consider these identities separately, Black feminist thought emphasizes the need for a holistic understanding of identity and power because Black girls face forms of discrimination and gendered racism that are different from what Black men or White women experience. Intersectionality, a concept rooted in Black feminist thought, highlights how overlapping identities such as race, gender, and class create unique forms of oppression or privilege for individuals that cannot be understood in isolation (Crenshaw, 1989; Shields, 2008).

Black girls face specific and unique challenges in their daily lives due to both racism and sexism, and one manifestation of this intersectional oppression is adultification. Epstein et al. (2017) noted that the adultification of Black girls in school settings can lead to harsher disciplinary actions, reduced empathy from educators, and a denial of much-needed support. Black girls are also more likely to be the subject of misinterpretation of mental health concerns, such as signs of anxiety, depression, and trauma, from adult figures, especially in schools, where they are punished for their actions instead of receiving needed support (Riddle and Sinclair, 2019). The adultification of Black girls occurs outside of school, where Black caregivers or family members provide more responsibility (e.g., caretaking, working to help with financial issues) to Black girls when they are not developmentally or emotionally ready to do so (Puig, 2002). However, engaging in these responsibilities can also be a protective factor for Black girls, as they can develop skills related to confidence-building and resiliency. While Black girls and women have been able to demonstrate joy and success when facing gendered racism, the idea of having to be consistently strong and resilient can sometimes undermine the dire need for additional support or lead to blaming Black girls for their adversities instead of blaming the oppressive systems causing the adversities in the first place (Bentley-Edwards and Adams, 2024).

##### 2.1.1.1 Black girls experience with social media

While previous literature has explored the increases in social media use and access among African Americans and girls (Lenhart, 2015; Rideout et al., 2022), few studies have examined the intersection of these social identities and their experiences with social media platforms. Studies focusing on Black girls’ experiences with digital spaces demonstrate that online platforms can be beneficial for Black girls; however, they are not without caution. According to Wade (2019), digital spaces can foster community among individuals, creating accessible support systems around the world. Social media, such as TikTok, can also serve as a space that encourages storytelling and counter-narratives (Nashid, 2024), which are strategies related to resistance and survival against dominant beliefs and values. Additionally, some Black girls are able to feel some sense of security and use digital platforms as respite from the racialized and gendered policing and discrimination that they experience in their daily lives (Wade, 2019). However, while Black girls have the ability to control who sees their digital content and who they engage with online, digital spaces, such as social media platforms, can still operate as sites of oppression.

Social media also has the ability to increase girls’ likelihood of engaging in self-objectification, where girls put more stock in their external appearance and believe that they are sexual objects due to pressure of having to keep up with Western beauty standards (Smolak and Murnen, 2011). While all girls may be exposed and pressured into self-objectification while online, Black girls tend to have a different experience where they are the subject of racial fetishism and gendered racial stereotypes and microaggressions (Gadson and Lewis, 2022). Black girls are burdened with the task of having to navigate a space that, within its core, is rooted in whiteness; however, Black girls are still able to use social media to exist outside of gendered racial perceptions and authentically be themselves by disengaging with whiteness and countering the narratives about Black girls (Nashid, 2024).

### 2.2 Limitations of methodologies traditionally used to study Black youth’s experiences

Due to the nuanced gendered racial experiences that Black girls endure in their physical and online communities, there is a need for further exploration of this topic in scholarly literature. However, research involving young people has often been shaped by methods that do not fully center their voices or lived experiences. Traditional research approaches tend to position youth as participants rather than as contributors to knowledge (Tilley and Taylor, 2018). In many studies, adults design questions, collect data, interpret the findings, and present conclusions without meaningfully involving youth in the process (Tilley and Taylor, 2018). This creates a power imbalance where young people have little control over how their stories are told and understood. Daley (2015) explains the consequences of excluding people from active roles in research:

The exclusion of people from research, in the name of “protecting” them, prevents research from being able to give voice to oppressed groups, and thus limits opportunities to advocate for change in these people’s circumstances (p. 13).

This exclusion matters especially when considering Black youth. Critical Youth Studies argues that researchers should reflect the rich, layered realities of young people’s lives rather than portraying them as fixed, one-dimensional, or defined by problematic behavior (Tilley and Taylor, 2018). However, academic and social institutions continue to ignore the perspectives of Black youth, especially Black girls, and when they are included in research, they are often portrayed in harmful ways. Sometimes they are seen as needing someone to “save” them from their dangerous neighborhoods, and other times they are treated as problems who threaten their communities (Ferguson, 2000). These limitations show the need for research methods that do more than just study Black girls. Furthermore, while qualitative methods can uplift voices and highlight intersectional experiences (Innis-Thompson et al., 2022), some may argue that this approach is inadequate (Agger et al., 2024). In addition to conducting qualitative data, researchers should also work with Black girls as authentic and meaningful partners. Research should recognize that Black girls, in particular, are not just people being studied, but experts on their own experiences within unfair systems. If we meet Black girls where they are and share power, they will have more agency to discuss issues that matter to them, explain their own realities, and help create knowledge that can bring real change to their schools and communities. One approach that does this is YPAR.

### 2.3 The need for YPAR when partnering with Black girls

Because traditional methods like interviews, observations, and focus groups cannot fully capture the complexity of young people’s decisions, actions, and lived experiences (Tilley and Taylor, 2018), approaches that center youth more directly in the research process are needed. YPAR addresses this gap. YPAR is a method that actively involves young people in investigating and addressing social issues that affect their lives by identifying problems, conducting research, reflecting critically on their lived experiences, and taking action to create change (Cammarota and Fine, 2008). Rather than adults controlling the research, YPAR focuses on collaboration and values the unique skills and contributions of young people as researchers (Rodríguez and Brown, 2009). The youth help decide the questions, collect information, interpret findings, and take action based on what they learn. The approach also aligns with refusal analytic procedures (Tuck and Yang, 2014), where the focus is not on Black girls as subjects of the study, but on sharing power as active researchers who examine the systems in their communities that perpetuate oppression or joy. The need for YPAR for Black youth is based on several key arguments: the opportunity to challenge systemic oppression, empower youth through leadership, and address issues such as community violence, school segregation, juvenile justice, and educational inequity (Cammarota and Romero, 2009).

For Black girls, YPAR provides a space to explore and respond to intersecting sociopolitical and structural challenges. It gives them the chance to examine and confront issues like race- and gender-based inequities and to develop a deeper understanding of their unique identities (Davis, 2020). For example, Hope et al. (2015) explored how Black high school students understood experiences of racial discrimination and inequality within their schools. Students were able to critically reflect on issues such as racial stereotyping, inequitable school environments, and limited institutional support. Additionally, Rose et al. (2024) highlighted the paucity of literature centering youth from racially and ethnically minoritized backgrounds who are impacted by oppressive systems and critically examined and reflected on conducting YPAR in academic spaces. Thus, more studies are needed that partner with girls impacted by systems of oppression and center their work on the impact of those systems (Foster, 2024; Rose et al., 2024; Tuck and Yang, 2014).

Few studies have used a YPAR approach to work with Black girls to explore their educational, physical, and social-emotional experiences in their communities and the action-based change that they facilitated. To inform a new community-based program focused on physical activity, body image, and self-esteem, Chard et al. (2020) collaborated with ten Black adolescents to create a leadership council using YPAR. Additionally, some studies specifically challenge the current state of educational systems, policies, and procedures while also exploring youth’s needs based on the intersection of gender and race (Davis, 2020; Foster, 2024). Black girls in a Southern U.S. alternative school setting engaged in YPAR to explore how exclusionary discipline practices, biased adult-student relationships, and limited access to supportive programming influenced Black girls’ educational experiences and emotional well-being (Troutman, 2026). Similarly, one YPAR study used a photovoice methodology to examine the gendered racial lived experiences of Black girls and college women at a predominantly white institution and their conceptualization of identity (Payne, 2023). Hence, by positioning Black girls as researchers, they gain the opportunity to learn through research that explores complex power relations, histories of struggle, and the consequences of oppression, and they can begin to re-envision and denaturalize the realities of their social worlds (Cammarota and Fine, 2008; Joseph et al., 2016).

For this study, the authors’ initial research questions focused on how sites of oppression shape the lives of Black students in a Southern city and how students can disrupt those sites (González et al., 2019). Using these questions to guide discussion, youth researchers in the YPAR study co-designed their own specific research question related to the authors’ initial research questions. The following research question was addressed by using qualitative methodologies such as photovoice and phenomenology:

*How does social media influence the adultification and sexualization of teenage Black girls in their local community?*

## 3 Material and method

### 3.1 Participants/youth researchers

The YPAR project was conducted in a Southern city located in the United States. In this city, the population consisted of around 320,000, of whom 14.5% identified as Black. The youth researchers in this study all attend the same public, suburban high school, which currently enrolls 1,770 students. Approximately 46% of students are eligible for free or reduced lunch. The school’s student racial/ethnic makeup was approximately 42.6% White, 30.3% Black, 16.6% Hispanic, 6.4% Two or more races, and 3.9% Asian. Additionally, as of 2022, female students made up roughly 53% of the student population. Eligible youth researchers included individuals: (a) aged 14–17, (b) who were enrolled students at a local partnering high school, and (c) who were current members of the Black Student Union (BSU). The youth researchers in this study were five Black, female high school sophomores (see Table 1). During the study, two student youth researchers disclosed that they were of Ghanaian descent. The pseudonyms for the youth researchers were created by using Tzioumis (2018) dataset on the most frequently used Black or African American names for girls.

**TABLE 1:** Youth researchers’ demographics and pseudonyms.

Pseudonym	Grade	Gender
Ayanna	9th	Female
Tomeka	9th	Female
Latanya	9th	Female
Ebony	9th	Female
Ivory	9th	Female

The grade reflects the current grade that the student completed prior to the summer break.

### 3.2 Recruitment

Because of an established partnership with a local high school’s BSU and the second author, this study used convenience and purposeful sampling for recruitment. Passive recruitment was utilized. Informational flyers describing the study, compensation, and risks were shared with the high school’s BSU director, who then disseminated the information with their students. The director did not answer any questions or recruit directly and sent all specific questions to the research team. Students chose whether to participate by filling out a confidential Qualtrics form that included the following questions: (a) Are you age 14–17 as of February 15, 2025? (yes or no), (b) What is your interest in racial justice research? (short answer), (c) What skills do you bring to a research team? (short answer), (d) Are you available to attend all 8 sessions? (yes or no), and (e) Do you have reliable transportation to [historic community center]? We used 17 years of age as the inclusion criterion because 18 is considered the start of emerging adulthood and to limit conflicts with summer schedules as seniors move on to their next endeavor after high school. Additionally, we could not provide transportation for this study, so we included a question about reliable transportation to the community center.

For the written responses to the short answer questions on the Qualtrics form, the researchers used a criteria rubric to help determine the eligibility of youth researchers. This was included in case more than five individuals were eligible for the study, as this study had funding restraints and wanted a small number of participants for a pilot study. For the open-ended statement, “Please briefly describe your interest in racial justice research/projects and why you would like to be involved with this project,” the adult researchers scored the responses of those who completed the form using the following categories: genuine interest in racial justice (i.e., does the student express sincere and thoughtful interest in the topic), personal connection or motivation (i.e., does the student mention personal reasons, experiences, or values that drive their interest), understanding of racial justice work (i.e., do they show a clear understanding of what this research involves and why it matters), openness to learning and growth (i.e., do they express curiosity, humility, or a desire to learn more), and communication skills (i.e., is the response clear, focused, and engaging). The scale scores for each category ranged from 0 to 3, with 0 representing a response that showed “no evidence or was off topic” and 3 representing “strong evidence of the quality.” For this question, students needed a total score of 11/15 or higher (at least 70%).

For the open-ended statement, “Please briefly describe what skills you bring to a research team,” the adult researchers scored the responses of those who completed the form using the following categories: relevant skills (i.e., does the student mention specific skills that would be helpful in a research team), teamwork and collaboration (i.e., does the student show they can work well with others and value shared ideas), problem solving or critical thinking (i.e., does the student show curiosity or problem-solving ability), and self -awareness and growth mindset (i.e., does the student reflect on their strengths and show willingness to grow). Using the same 4-point rating scale rubric, students needed a total score of 9/12 or higher (at least 75%) to be eligible. All individuals who completed the screener became youth researchers of the study.

### 3.3 Setting

This project took place at a historic local community center that serves people in the high schools’ zoned area and is located in a predominantly Black neighborhood. The community center was established in the 1930s as one of the first public recreational facilities built exclusively for Black residents, and it has served the community for nearly a century through youth programming, mentoring, and community workshops and events. The center’s historic facility, resources, and supportive staff allowed the young people to engage fully in the project, share their experiences, and take part in research activities without feeling judged or restricted. The community center is open to outside organizations and parties to host ongoing programs in its meeting rooms, and it approved this study, via an application form, to take place during the summer months.

### 3.4 Positionality

The first author is a Black, American, heterosexual, cisgender woman who is an Assistant Professor of School Psychology at a research-intensive university in the South. As a Black woman who was not so long ago a Black girl, she has had experiences in her own life where she was exposed to adultification. However, she is dedicated to recognizing her own biases and acknowledges that her personal experiences may differ from those of other Black girls. As a researcher, her work aims to center the experiences of Black youth by empowering them to be the authors of their own stories and facilitators of change. While she recognizes that she holds a position of power as an academic, she also sees this position as an opportunity to decolonize traditional research practices with racially and ethnically minoritized youth, enabling them to share their life experiences and discuss solutions in their communities.

The second author is a white, queer, cisgender, middle-class woman born and raised in the United States who is a current Clinical Assistant Professor in Secondary Education at a Southern R1 university. She developed a partnership with the BSU at the local high school 2 years prior to the study and worked with all five co-researchers during their freshman year. Her work has interrogated the role of white women in the fight for racial justice (Doyle, 2018), and she recognizes the honor and sacredness of being trusted by her co-researchers and colleagues to be a part of this project. Throughout the research project, she navigated the tension between her facilitation experience as a former high school teacher and the necessity of not overtaking a conversation about racism that is not her lived experience–explicitly naming this to the youth researchers at the outset and asking questions rather than naming things for the students.

The third author is a Black, American, heterosexual, cisgender woman who is a doctoral candidate at a research-intensive university in the South. Like the first author, she has had personal experiences with the adultification of Black girls. However, she entered this study with an open mind, curious about what the participants had to say about the research rather than making it about her own experiences. Additionally, as a former early childhood educator, she has seen how social media has affected younger Black girls and was excited to give these youth researchers space to voice what is important to them and how social media has affected them. This is especially the case since the youth researchers have grown up with technology and social media from a young age, unlike the third author, who did not when she was growing up.

The fourth author is an African, heterosexual, cisgender woman, as well as an immigrant and international doctoral student. Her cultural background shapes how she understands the experiences of Black girls. She recognizes that perceptions of Black girls as more mature extend beyond the United States and may influence how their behavior is interpreted in different contexts. Drawing from her experience as a former educator in an Asian country, she has witnessed firsthand how Black girls are adultified across various spaces.

### 3.5 Research design

#### 3.5.1 YPAR approach

Youth Participatory Action Research is a framework that positions young people as experts in the research process rather than participants, and it centers youth as change agents for social justice initiatives (Caraballo et al., 2017). In this study, five youth researchers who were involved in the research process and were compensated for their time and expertise. This YPAR study utilized qualitative methods as its primary data collection approach to explore the lived experiences of the youth researchers navigating social media platforms.

#### 3.5.2 Photovoice

Photovoice is a research method that allows individuals to document, explore, and share their experiences through photography (Wang and Burris, 1997). The youth researched utilized the photovoice methodology, with the adult researchers facilitating the process; it provided a way to engage in refusal analytical procedures, as the youth researchers’ photos and captions provided an accurate representation of their thoughts and feelings. It also gave the youth researchers the power to determine which data would be included and which should not be privy to academic audiences. This methodology allows youth researchers to bring unique perspectives, foster discussion, and reflect on their individual thought processes (Wang and Burris, 1997). In this methodology, there are three goals: (1) to allow for researchers to easily record and analyze their community, its needs, and its strengths, (2) to foster deep discussion within the research team centered around the photographs, and (3) to reach those within the community and those with the power to make community change, such as policy makers (Wang and Burris, 1997). Each youth researcher was assigned an iPad to use for data collection. After taking a picture with their iPad, the youth researchers uploaded the photo, a caption, and a description to a confidential Qualtrics form labeled as their Photovoice journal. Before taking photos, the youth researchers were trained in photography ethics and how to take photos that go beyond the surface level, using Rutger’s Photovoice Facilitator Guide (Jongeling et al., 2016).

#### 3.5.3 Sessions

Using a YPAR approach, adult researchers facilitated eight structured, group YPAR workshops with the youth researchers (see Table 2). All sessions were informed by the YPAR Hub, available online through the University of California, Berkley, and the resources it provided, and lasted approximately an hour and a half. Each session began with an identity-based or community-building activity. This type of relationship-building lessened the typical, unspoken power dynamic between youth researchers and adult researchers (Young et al., 2024). The first two sessions allowed the adult researchers to gauge the youth researchers’ knowledge before the study began. This helped the adult researchers better tailor their approach to facilitating discussions and explanations of concepts to the youth researchers. Additionally, during the first two sessions, the adult researchers explained the research process to the youth researchers, including how to narrow possible research topics, how to create a specific research question, an introduction to photovoice as a methodology, and how to collaboratively come up with action steps as a group to achieve group research goals.

**TABLE 2:** YPAR session sequence.

Session	Activities	Description
Session 1: introduction to research and research question	Activity 1: icebreaker (identity/personality-based) Activity 2: research experience reflection Activity 3: video reflection [“Stand Up”] Activity 4: selecting a research topic	Youth researchers engaged in an identity-based icebreaker, shared their previous experiences with research, were introduced to the research process through the discussion of a YouTube video titled “Stand Up,” and began thinking of potential research topics.
Session 2: research question and research plan	Activity 1: icebreaker (name the group) Activity 2: what makes a good research question Activity 3: identify team goal and next steps Activity 4: introduction to photovoice	Youth researchers collectively chose a name for the group, discussed what makes a good research question, and learned how to develop clear and meaningful questions for their project. They discussed the areas of concern they want to address and the next steps for their research, including how they might collect information. They were introduced to photovoice, watched a video about it, and learned about ethical considerations when taking pictures for research.
Session 3: analyzing data part 1	Activity 1: icebreaker (create group expectations) Activity 2: review research topic, question, goals Activity 3: introduction to SHOWeD Activity 4: data sourcing handout	Youth researchers created group expectations through discussion. They narrowed and refined their research topics. The SHOWeD method of photo analysis was introduced. They examined existing data related to their research question and their own collected data (i.e., photographs and captions written in take-home journals). Then, they determined action steps based on what data they felt was missing or important to spread throughout the community.
Session 4: analyzing data part 2	Activity 1: icebreaker (team-building) Activity 2: discussion of initial themes Activity 3: SHOWeD worksheet Activity 4: SMART goal creation	Youth researchers began with a team-building activity called the “bag of stories.” During this activity, a bag with random objects was passed around, and each participant picked one item without looking. The group then created a story together, with each person adding to the story based on the object they picked. They discussed the initial themes from the previous week’s conversation by reviewing deidentified transcripts and identifying pertinent ideas. They used the SHOWeD worksheet to analyze the photos they had taken, write captions, and discuss what the photos revealed about the adultification of Black girls in their community. They collectively identified patterns in the photos and captions, created SMART goals, and discussed possible action steps.
Session 5: analyzing data part 3	Activity 1: review group expectation and icebreaker Activity 2: theme “Tracker” Activity Activity 3: affinity-mapping / grouping with participant pictures [SHOWeD] Activity 4: action steps toward goals	The youth researchers reviewed the group expectations and took part in a “Two Truths and a Lie” icebreaker. They read a short fictional story and shared words, phrases, or feelings that stood out to them. They used the SHOWeD worksheet to analyze the photos they had taken, write captions, and share the meaning behind their images. Together, they discussed patterns and themes that appeared in the photos and connected them to their research questions. The group then discussed action steps toward their goals.
Session 6: applying research findings	Activity 1: icebreaker (community-building) Activity 2: “Data for Change” handout and discussion Activity 3: action steps (create a plan)	Youth researchers began with a community-building icebreaker activity. They reviewed the “Data for Change” handout and discussed how data can raise awareness and create change in the community. They discussed the type of change they hope to see and the skills and resources they can utilize to achieve it. They ended the session by creating a plan for how they would use their data to promote change in their community.
Session 7: sharing your story with the media	Activity 1: icebreaker (community-building) Activity 2: member checking Activity 3: project story and impact planning Activity 4: individual action steps	Youth researchers engaged in a community-building activity that involved member checking. The transcript of the previous session, in which the youth researchers discussed their themes, was printed; They highlighted important quotes and related topics and themes. Adult researchers explained to youth researchers the importance of publicity and spreading their findings and co-created an action plan with specific tasks for individuals.
Session 8: sharing our story	Activity 1: icebreaker (community-building) Activity 2: member checking Activity 3: creating content Activity 4: reflections	Youth researchers engaged in a community-building activity. They reported the status of their action steps from the previous week. They made initial content for their social media account and planned for the community event. Youth researchers then reflected on their experience with the research study and completed a final review of the transcripts and codebook.

Sessions three through five focused primarily on the youth researchers collecting data within the community, through photographs taken on iPads, identifying themes, and applying those themes to their original research question. In these sessions, the youth researchers were introduced to the See, Happening, Our Lives, Why, Educate, Do (SHOWeD) method of analysis, a structured process used in photovoice to guide critical discussion of photographs. Youth researchers selected photographs from their collection and completed a worksheet outlining these questions. This process helped them explain their photographs and tie them back to their themes and initial research questions. In session five, the youth researchers engaged in a theme “tracker” activity, in which a scenario pre-created by the adult researchers was written on a whiteboard about the policing of Black girls’ behaviors and appearances in community spaces (e.g., schools). The scenario was based on existing literature on sexualization and adultification and served as a primer to familiarize them with theme development before they categorized their own photos. The youth researchers shared words, phrases, and feelings that came to mind while reading the scenario and discussed these ideas collectively by providing annotations. Then, the youth researchers engaged in an affinity-group/mapping activity and created an initial list of themes (Figure 1).

**FIGURE 1:**
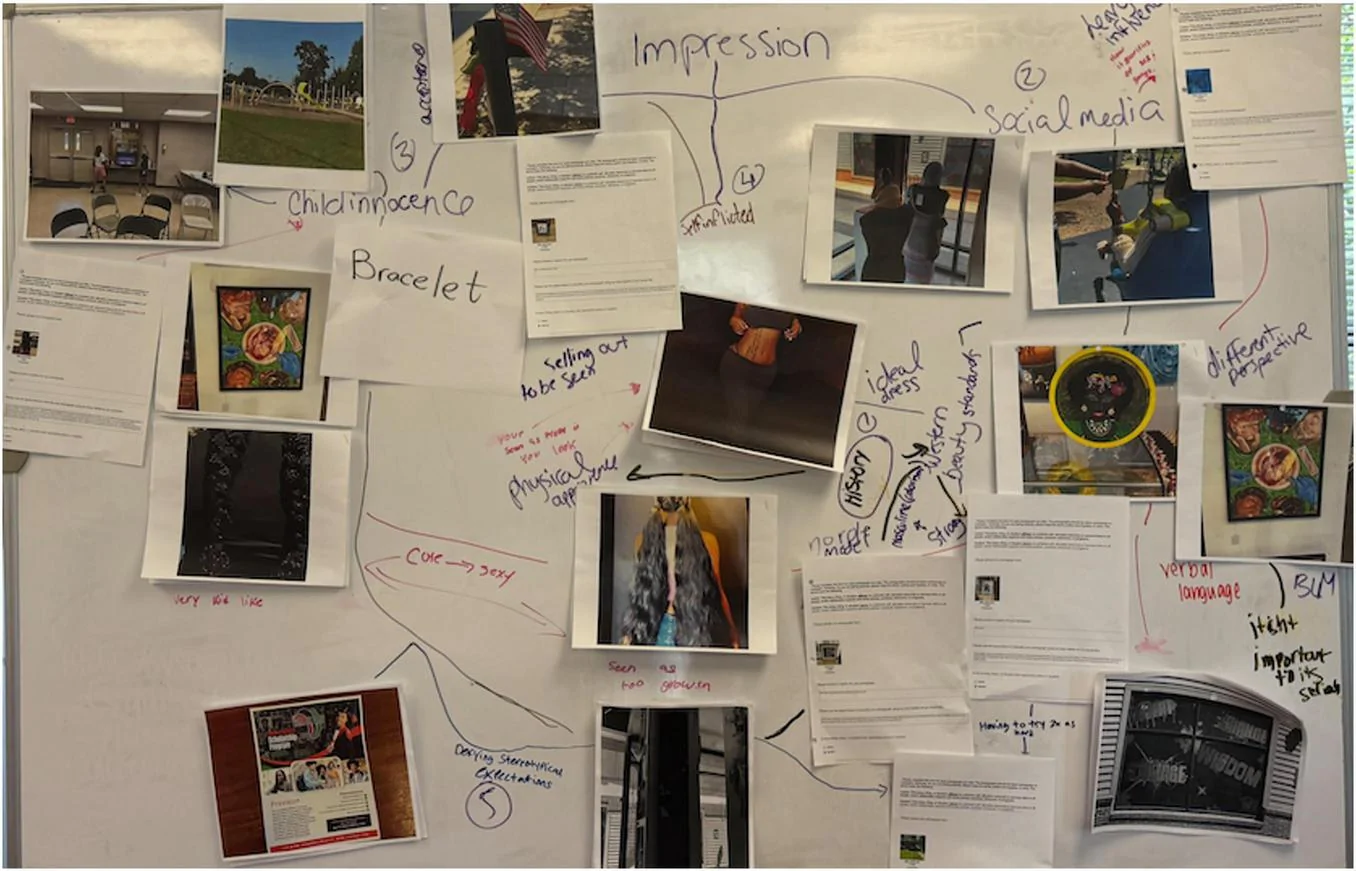
Image of the affinity group/mapping activity.

Sessions six through eight focused mainly on the youth researchers’ understanding that their collected data was important and that the data and their findings should be disseminated throughout the youth researchers’ community. In sessions six and seven, the youth researchers discussed their target audience, the best ways to share their information and findings with that audience, and which tasks each individual should do, leaning into individual strengths. After agreeing to create a social media page and interview community members at a community event, in session eight, the youth researchers finalized their plans to share their findings and recorded social media content related to their findings and the community event.

### 3.6 Data analysis

For this part of the study, the youth researchers analyzed all data related to their research questions in three stages (Wang and Burris, 1994). In stage one, youth researchers decided which photographs closely aligned with and answered, in their opinion, their original research question: “How does social media influence the adultification and sexualization of teenage Black girls in the South?” Each youth researcher wrote captions for their photographs. In the second stage, they provided the group with contextual information about their photographs, reflecting on why the photographs related to the research question. This was done through the SHOWeD method of analysis (Wang, 1999). SHOWeD is a structured reflection process that guides participants to critically analyze and explain the meaning behind their photographs by asking them six questions: (1) What do you see?, (2) What is happening here?, (3) How does this story affect our lives? How does it make you feel? What does it make you think?, (4) Why are things this way?, (5) How could this photo educate people?, and (6) What can we do about it? (Wang, 1999). Lastly, in the third stage of data analysis, the lead investigators guided the youth researchers through an affinity group/mapping activity, which produced an initial list of themes through thematic analysis. Affinity mapping is a qualitative analytic technique used to organize large amounts of qualitative data by grouping related ideas into categories based on natural relationships and patterns (American Society for Quality, n.d.; Wang and Burris, 1994, 1997). The adult researchers printed all the youth researchers’ photographs and tasked them with developing themes by grouping related pictures and captions during the YPAR sessions.

While the first two authors led the activity, the youth researchers used a whiteboard to tape their photographs and draw connections between groups of photographs to find common themes and relationships. Once the affinity grouping activity concluded, the youth and adult researchers discussed the initial themes (i.e., body type, child innocence, impressions, beauty standards, role models, self-infliction, and colorism) and their connection to the youth researchers’ initial research question. This list of initial themes was used as the initial codebook for a secondary analysis (Patton, 2002).

Each session of the current study was recorded, and two graduate students transcribed and cleaned the session transcripts for each week. As it relates to the youth researchers’ research questions, the transcripts from the affinity grouping session, photos, captions, and the SHOWeD data were analyzed in a secondary analysis to determine whether any new themes emerged after the affinity mapping activity. While triangulating the data, the lead authors used thematic analysis (Braun and Clarke, 2023), and *in vivo* coding was used to finalize the codebook. This analytic approach helped emphasize and maintain the voices of the youth researchers by developing and revising new codes based on their session artifacts (Malorni, 2025). The last two YPAR sessions included member-checking activities that allowed the youth researchers to provide feedback on two transcripts. However, due to time constraints in the summer, the first and second authors reviewed and coded the remaining data and met regularly to ensure consistent interpretations and address any queries. This process helped maintain the voices and perspectives of the youth researchers while also allowing the researchers to examine the data more closely.

## 4 Results

Five key themes emerged from the data related to the youth researchers’ research question regarding the influence of social media on the adultification and sexualization of Black teenage girls in the South: (1) the juxtaposition of childhood innocence, (2) lack of ideal role models, (3) the effect of other people’s actions, voices and opinions, (4) western beauty standards: us vs. them, and (5) the overlooked impact on mental health. The themes are listed in no particular order because they represent and emphasize the different perspectives and experiences shared by the youth researchers during the Photovoice process.

### 4.1 The juxtaposition of childhood innocence and adultification

The youth researchers discussed how perceptions of activities and behaviors can change instantly. They noted that some of the same behaviors and activities they engaged in when they were young were once deemed innocent; now, others view them as more mature. For instance, Ebony described this shift in a single image (Figure 2) featuring a young girl with colorful hair and beads. She notes that nowadays, if you see a young girl with this hairstyle, it is age-appropriate, but if you wear this style as a teenager, you are acting more like an adult:

**FIGURE 2:**
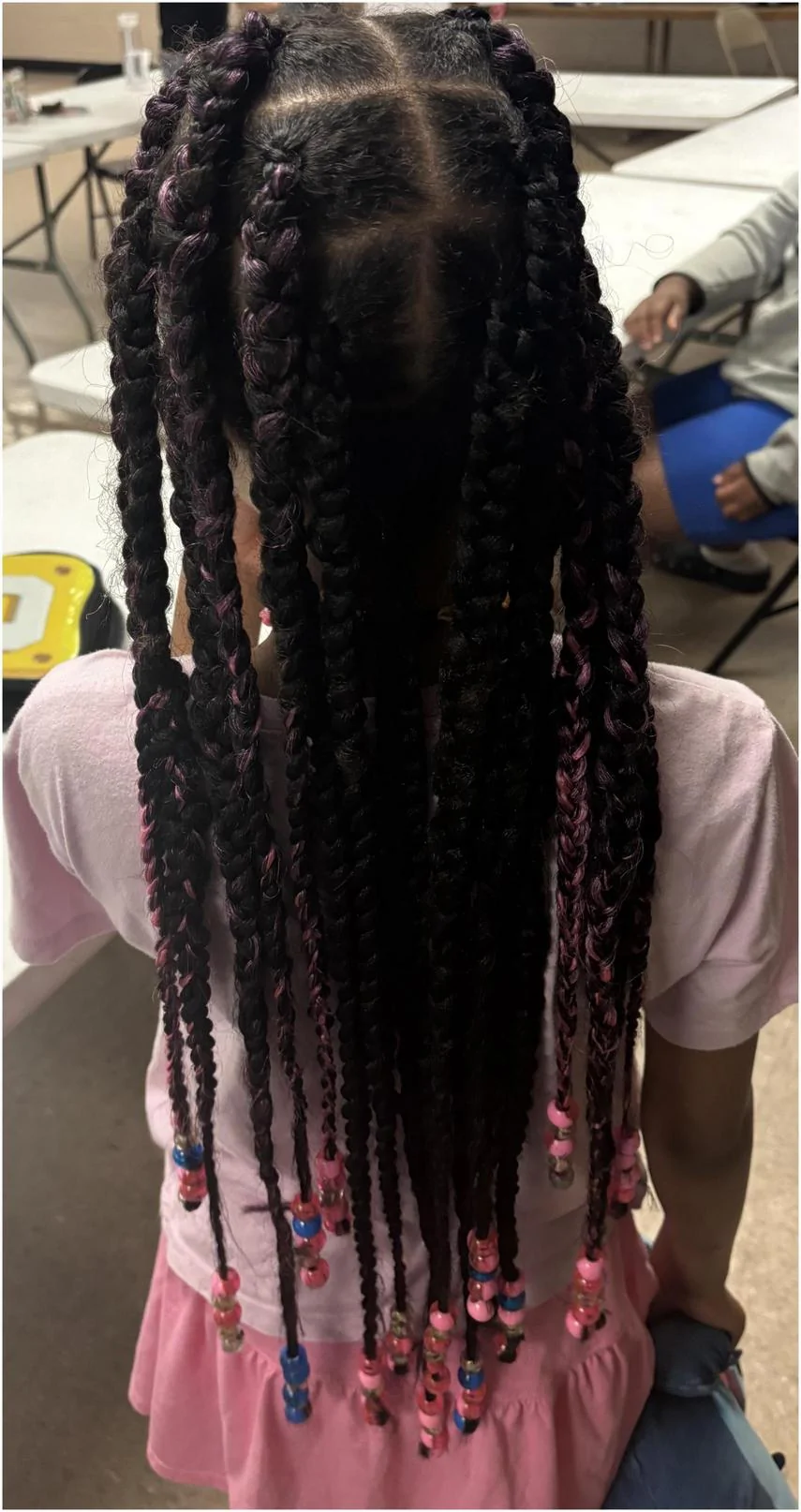
Girl with color hair and beads.

If you have a picture like this, you’re not seen as too grown. It’s like, oh, cute. Like it’s a cute little hairstyle. Now if you wear this, you’re, oh, you’re kind of too grown cause it’s not braids anymore. It’s more like long, wavy Brazilian bundles…Yeah, like there’s a difference in how we’re seen because if you wear this, you’re more grown up. You’re trying to act more like an adult. If you wear a style like this, it’s more like, okay, you’re still kind of a kid, but you just got like braids. Okay, you’re cute, or whatever. So, I made that connection.

Another youth researcher, Ivory, explained the meaning behind one of the images (Figure 3), which showed a child on the swings being shown a phone. Ivory shared that children should be allowed to play freely on playgrounds, but many are being exposed to social media and the internet at a young age. This early exposure can impact how Black girls view themselves and how they should act.

**FIGURE 3:**
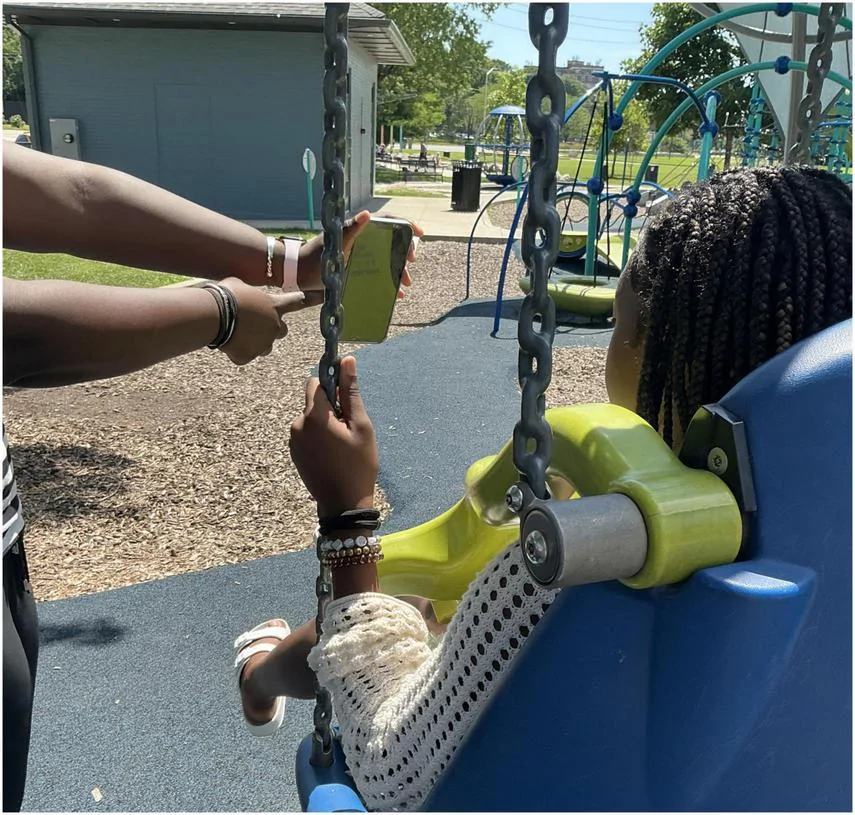
A young Black female child being forced to look and be consumed by the media.

To support the idea that Black girls are being exposed to social media at an early age, another youth researcher, Tomeka, took a picture of an empty playground (Figure 4) and added the following caption:

**FIGURE 4:**
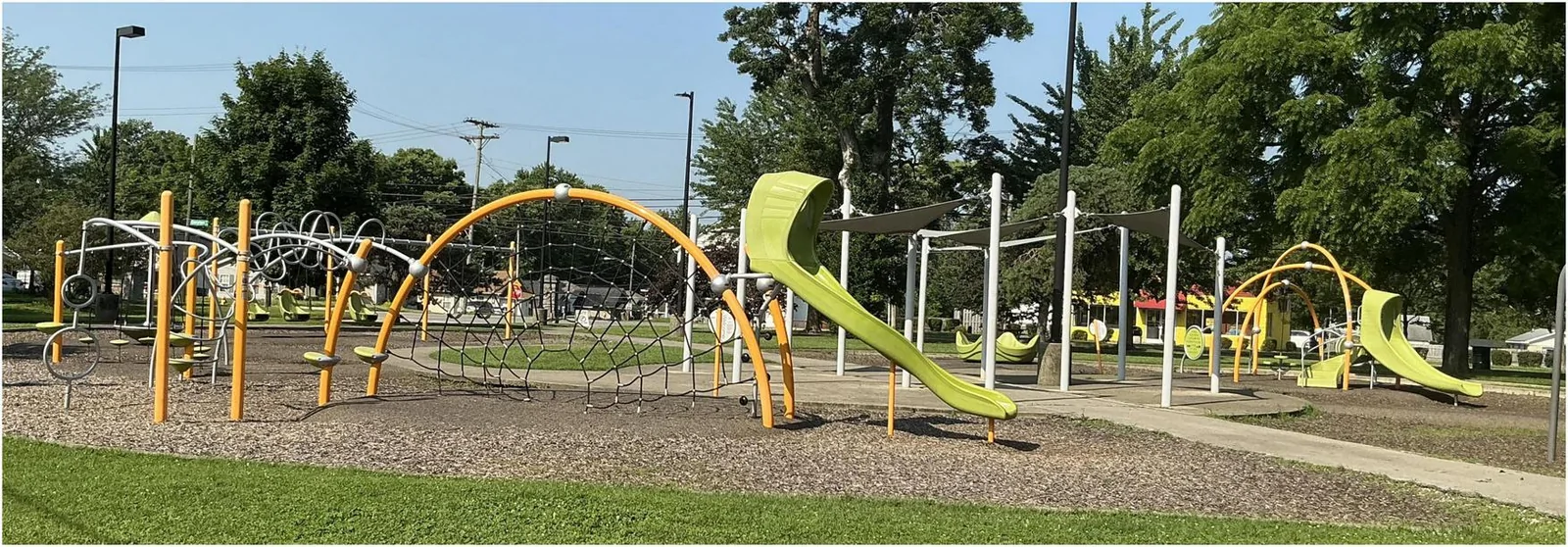
Empty playground.

This is a picture of an empty playground. I took a picture of this because I wanted to show the separation and distinction between black girls and childhood activities. Usually, you would see the playground full of kids, playing with each other or playing by themselves on the swings or slides, building connections with the playground or other kids, but with the adultification of these girls, causes them to distance themselves from what teenagers or kids usually do, because they are so concerned with growing up, especially when getting a new phone or getting boyfriends or friends that lead them down opposite roads.

The youth researchers acknowledged that dancing is now part of social media and that some dances are hypersexualized or feature explicit songs in the background. They noted that when Black girls are dancing, they are often adultified. However, Ebony attempted to challenge that narrative by discussing a picture (Figure 5) of Black girls dancing at a community center:

**FIGURE 5:**
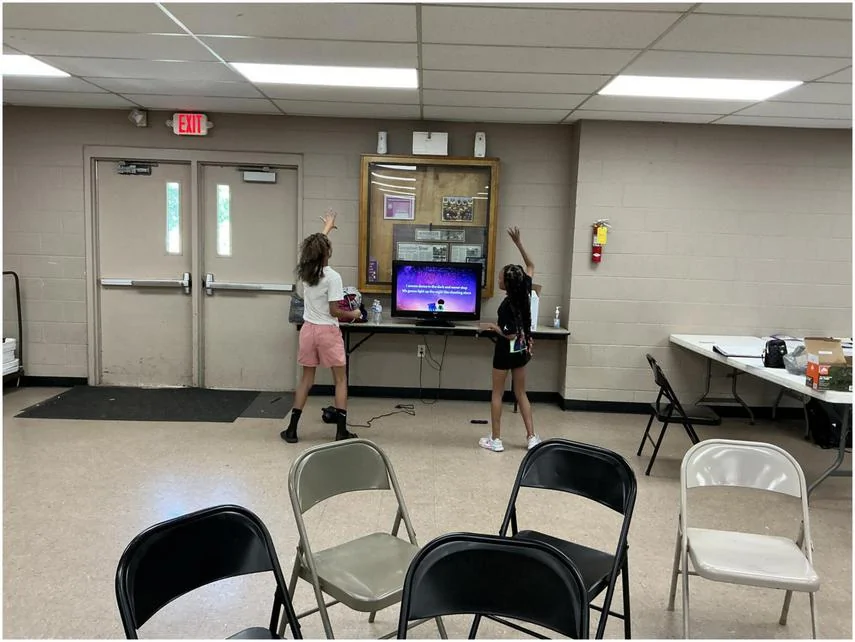
Freedom.

I took a picture of these two little girls dancing to “Dancing in the Dark” by Rihanna. This song is very carefree and childish because it was seen in a children’s movie. I feel like this song advocates freedom for everyone, even if they are different, and I feel like the dance that the kids are doing really helps express that feeling. It represents how these young black children can break away from the stereotypical ideas that people have about black people or young black girls, and I feel like this really helps understand that not all black girls want to be sexualized or mature just yet but want to live in the present moment and live with their childlike self.

### 4.2 Lack of ideal role model

The youth researchers agreed that, as they got older, they expressed that Black girls do not have a lot of Black women to look up to as role models. The majority of youth researchers emphasized the historical teaching of white role models or specific Civil Rights leaders, such as Rosa Parks, but expressed the need for more exposure to Black women who have done so much for this country. One participant, Tomeka, noted that the lack of Black women to look up to causes Black girls to look up to “bad role models” and desire to be just like them. Furthermore, while discussing the topic of limited role models, the youth researchers noted famous, prominent Black women rappers who are consistently shown on social media, and the representation of Black women:

At the end of the day, if we are trying to bring ourselves up, Sexy Red, Cardi B, Nicki Minaj, even Megan, I love them, but like, I have to say, like about anything else except for like your body or like sex.

Like, is that all we have to offer?

To add to the youth researchers’ perception of how Black girls are represented, they also discussed how stereotypes of Black women and girls are never addressed within or outside of their community. Ebony displayed a picture of a flyer of young Black girls (Figure 6) and expressed the following:

**FIGURE 6:**
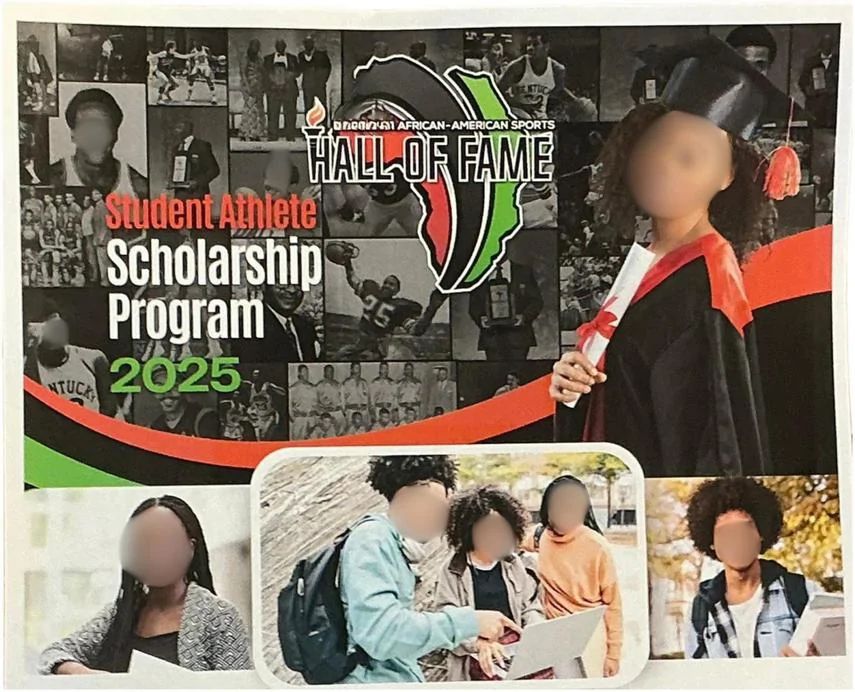
More than capable.

The flyer includes pictures of young Black girls, portraying them in an academically successful and achieving manner, which is the opposite of what the media often portrays [them] as–ghetto, nasty, ratchet, and unprofessional.

After sharing this image in a large group, youth researchers expressed a need to problem-solve and address current, negative narratives about Black girls. The group discussed how to reimagine the image of Black girls in a more positive light and to recognize the many possibilities Black girls can achieve.

Instead of seeing Black women in a horrible picture or [displaying] horrible mannerisms, you see them like in schools, and they’re like graduating…So it [the picture] was like showing that Black kids, Black women can do that too.

### 4.3 The effect of other people’s actions, voices, and opinions

The youth researchers shared that Black girls are no longer focused on being teens and tend to behave more like adults, especially in their clothing. During this discussion, Tomeka provided a reason for why she believed this was the case, noting that “Kids are very impressionable–so whenever younger kids are seeing that type of thing, they’re thinking in their mind, I can be like this too, and people will like me.” At the same time, other youth researchers noted that some individuals [influencers] promote the idea that appearing and acting older is considered beautiful. Additionally, while referencing an image of a flower with butterflies (Figure 7), Latanya noted that the picture represents the power and influence of social media. Latanya then went on to share that the image highlights how Black girls think that other people only want to be around those who are mature. The youth researchers also noted that there is a never-ending cycle regarding the analysis and interpretation of other people’s comments on social media.

**FIGURE 7:**
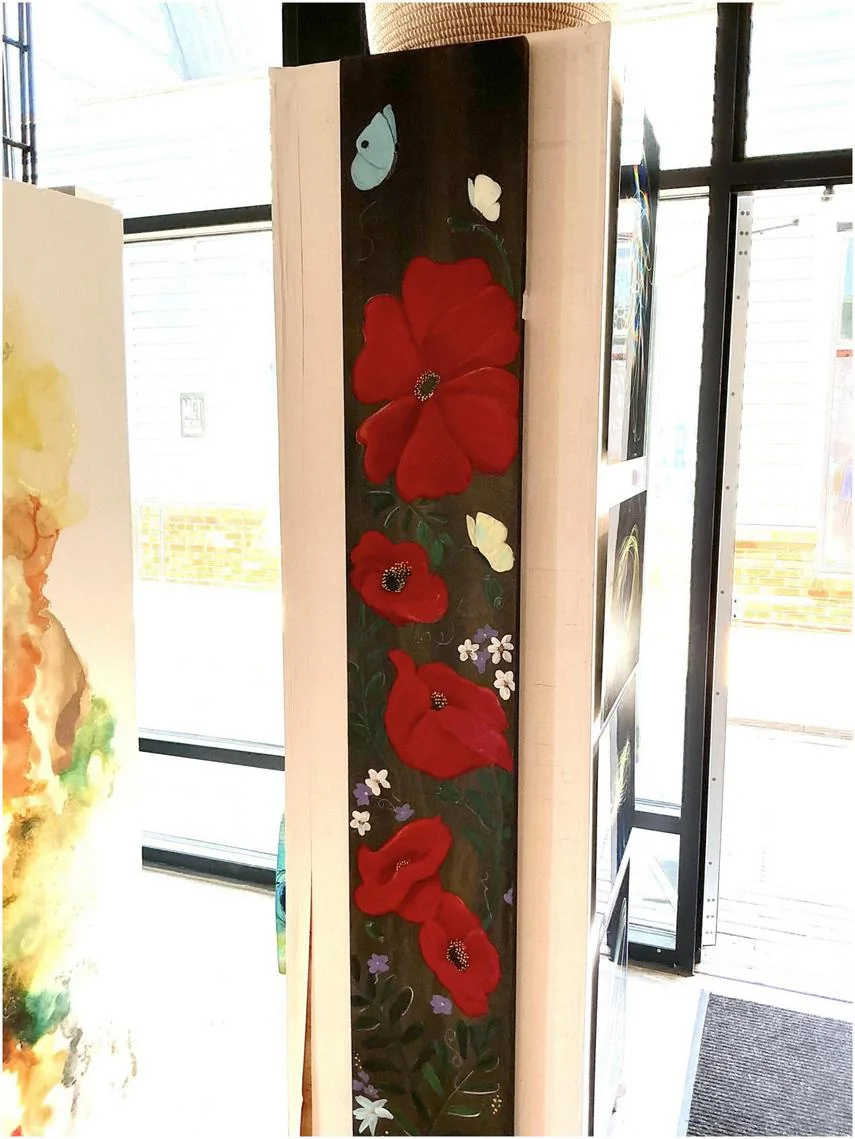
Bloom.

But at the top of the bloom flower, there were like butterflies like swarming, and I basically represented that to be like the audience. Because I feel like the audience, they swarm more to people who act more mature, even if they aren’t ready to be mature yet. And so I felt like that was a correlation between childlike innocence and social media influence.

Youth researchers also discussed how same-age peers can encourage some girls to change their appearance to meet the expectations seen on social media:

12-year-olds act like 12-year-olds, and they get made fun of…When I was an eighth grader, we had like fifth graders come in. I was walking the hall at one time, and this girl, she looked like a sixth grader…She looked cute, and I was passing by to go to the bathroom when her friend was like, “You need to dress more like a 6th grader.” And in my head, I was like, how does a 6th grader dress? It’s not like she had like a polo, a Walt Disney-like hoodie on or whatever…So I’m like, how do you want her to dress if this is not 6th grade like, you know?

Youth researchers linked the pressures of social media to being reinforced in the home setting. They shared that Black girls have a desire to replicate what they see on social media in terms of their appearance and behavior; however, this behavior is being reinforced as adultification by close adults. For instance, Ayanna used an image of two birds (Figure 8) to describe how close families can perpetuate the adultified narratives of the youth researchers:

**FIGURE 8:**
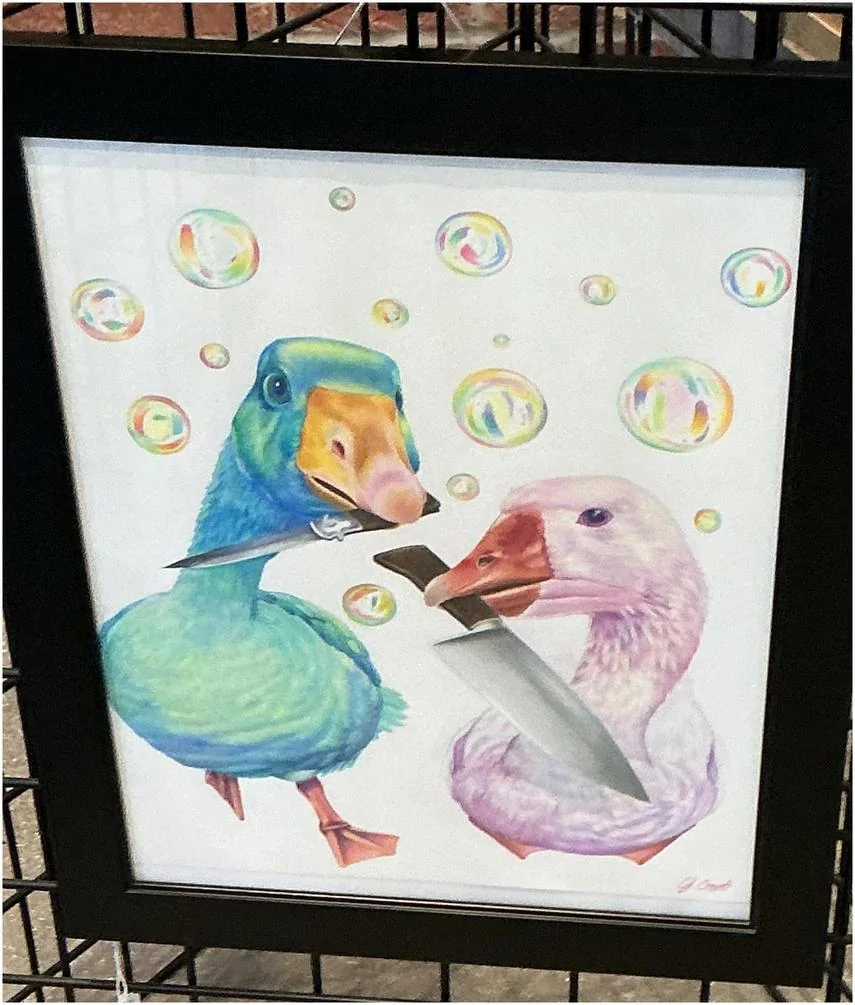
Are we killing each other.

A lot of adultification happens from inside the home, from parents telling their kids to change into appropriate clothes…the sad part [is that] some parents just want to protect their kids, but don’t know that they are also contributing to the problem.

### 4.4 Western beauty standards: them vs. us

While youth researchers noted social media pressure about how they dress, they also noticed similar pressure around their physical appearance, such as their hair. Hair and other physical attributes have historically been topics of discussion in relation to beauty standards in the United States. These attributes can be used to highlight European physical features as the “golden standard,” and anything that deviates from that is not considered beautiful, which affects Black girls and women. During the YPAR sessions, the youth researchers shared discourse about having to look a certain way to feel seen or desirable. They noticed how difficult it is for Black girls to experiment with their hair without being seen as attempting to look older. The youth researchers, more specifically Tomeka, Latanya, and Ebony, inferred that they often see different hairstyles on social media that they like; however, when they want to wear those said styles, there is often pushback. For example, Ebony outlined their frustration about attempting to wear different styles by describing an image (Figure 9) with a girl with braids:

**FIGURE 9:**
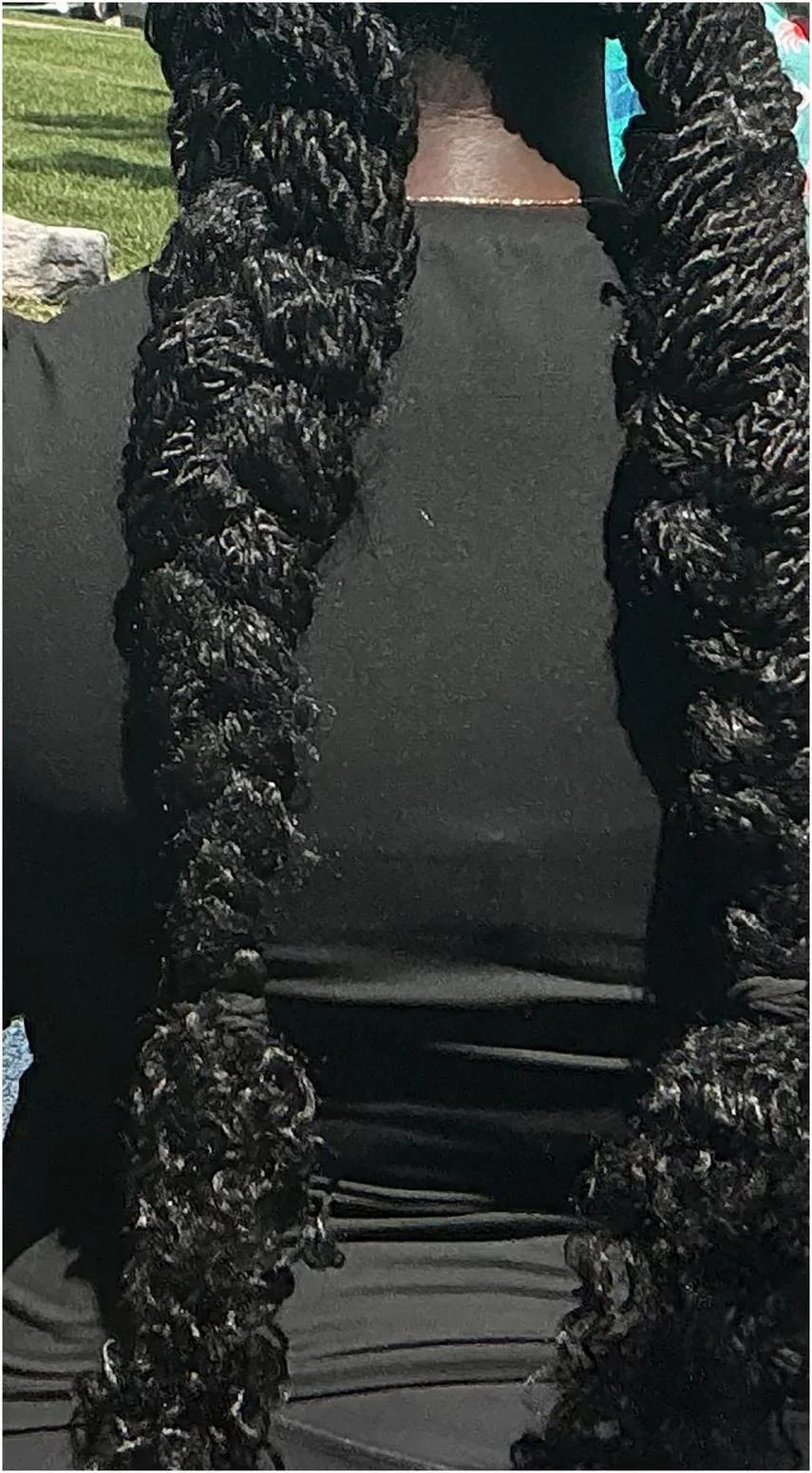
Hair getting attacked.

If you have braids in, then you’re being childish and not grown-up. If you have long wavelength bundles in your hair, you want to be grown and need to stay in your own age category. If you have a perm, you don’t love your natural hair. If you have your natural hair out, you need to get it done because it makes you look messy and unkept. Black women could never win when it comes to how they do their own hair.

Along with the pressure to meet beauty standards for their hair, the youth researchers also discussed the need to look a certain way to feel accepted in the United States. They emphasize that Black girls often have to be careful about their appearance and have to try extra hard to be accepted when compared to peers of different races but are often still seen as less. Furthermore, they also see other Black women and girls, who are social media influencers, being treated the same way regardless of their appearance; thus, implying that Black women can’t do anything without being judged and scrutinized by people online. One youth researcher, Tomeka, described a time when they noticed the double standards when it came to appearance online:

In social media, like it was gendered racist stereotypes, and I don’t know why, but for some reason, it made me think about prom season. And TikTok during around prom season, like some people are talking about how it was ghetto and like out of the ordinary… cause whenever you look at like a Black girl and what she wears, it’s always big and gold and shiny and colorful, that’s literally it, that’s the dresses they wear. And so they [individuals of other races] were like “ghetto prom,” but…it wasn’t like some white girls dressed like that, too. But nobody was in their comments calling them ghetto and like, “oh, you’re doing too much, it’s just a dance.” And I don’t know why, but it made me think…why is it that it’s a problem that they [Black girls] are enjoying themselves and they want to be pretty…but it’s not a problem when another group of people does it?

Ivory described an image (Figure 10) of eagles to show the perceived maturity level of Black girls compared to their peers. She noted how Black girls who are adultified also have protective characteristics:

**FIGURE 10:**
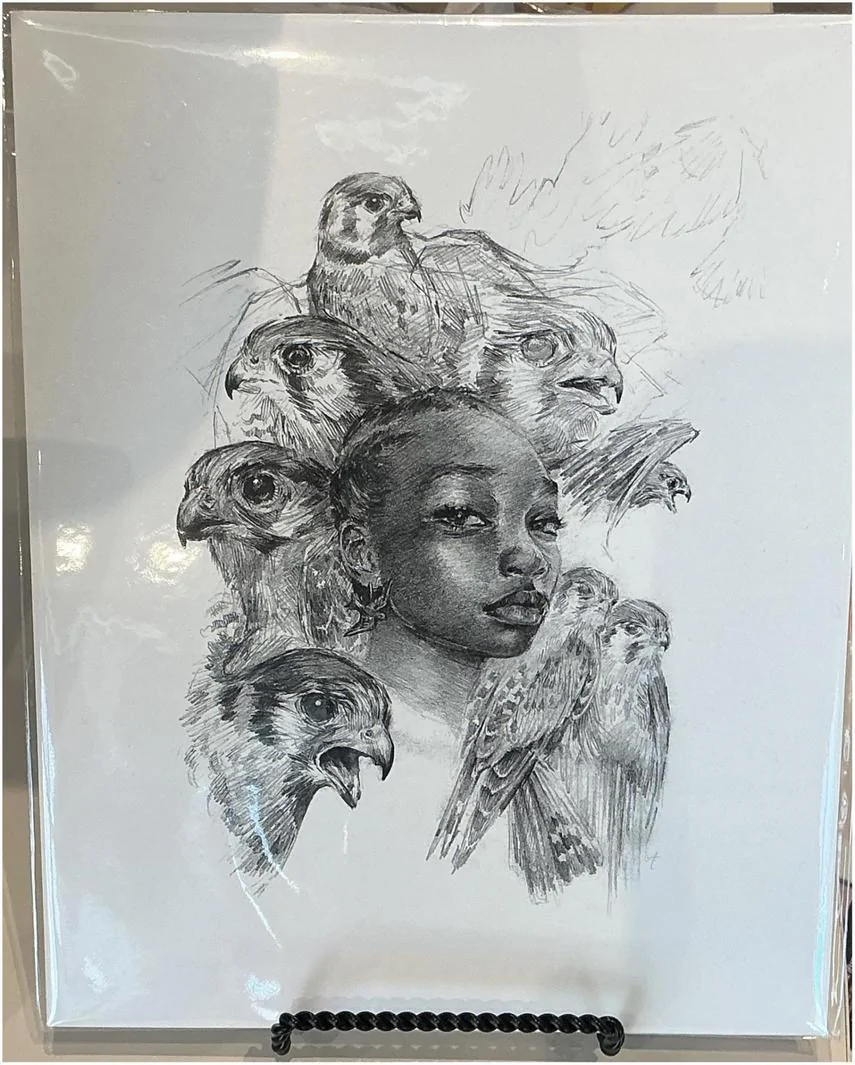
Protector.

Like eagles, young Black girls are seen to be fierce, more (emotionally) mature, and always protecting others, when compared to other young non-colored girls. Young Black girls often don’t get to be the kids they are, as they’ve always had to protect themselves and try twice as hard as their non-Black peers.

Youth researchers also shared that judgment about their physical appearance can come from their own communities. They noted that Black people contribute to the discussions about colorism in the Black community and reinforce adultification. They clarified that from their own experiences, women and girls with darker skin are seen as more masculine and get a lot of hate on social media, thus potentially motivating these women and girls to be more sexual for acceptance.

I know that darker skin, dark people are getting hated on, but like your own people, especially like your opposite gender, the people that are supposed to be there to protect you and stuff are calling you manly and this and that. That also gives the rights for other races to come in and continue to bash because they’re like, you know, your people are talking to you like that, so I can too.

Tomeka provided their opinion on how colorism on social media can impact the behavior of Black girls:

Social media pushes the fact that, oh, darker women are seen as more masculine…I think on social media, we can relate it [colorism] to body types and like body proportion, and how the way that people might not be seen as pretty or sexy can influence the clothing they wear and the way they act, or like the way they want to change their bodies just to look better for other people.

### 4.5 The overlooked impact on mental health

Youth researchers expressed concerns about how social media can affect youth’s mental health, with many of them agreeing that mental health is often overlooked and can motivate kids to continue to act older like the individuals they see on social media. They discussed how they would like adults in their lives to check in more often to make sure they are mentally okay, noting that adults often forget that kids can have feelings shaped by their virtual and in-person life experiences.

And people like overlook it [mental health] because they’re like, oh, you’re a kid, you don’t have these kinds of feelings, or like you’re not at the age where you would have these kinds of feelings.

Oh, you can’t have stress as a kid.

Yeah, you can’t act like this until you have a job, a house, all that stuff.

Crazy. Like, this is all stress to me. I should be like, stressed.

Ivory also described an image (Figure 11) of three similar garbage bins as a way to depict how Black girls feel compared to others:

**FIGURE 11:**
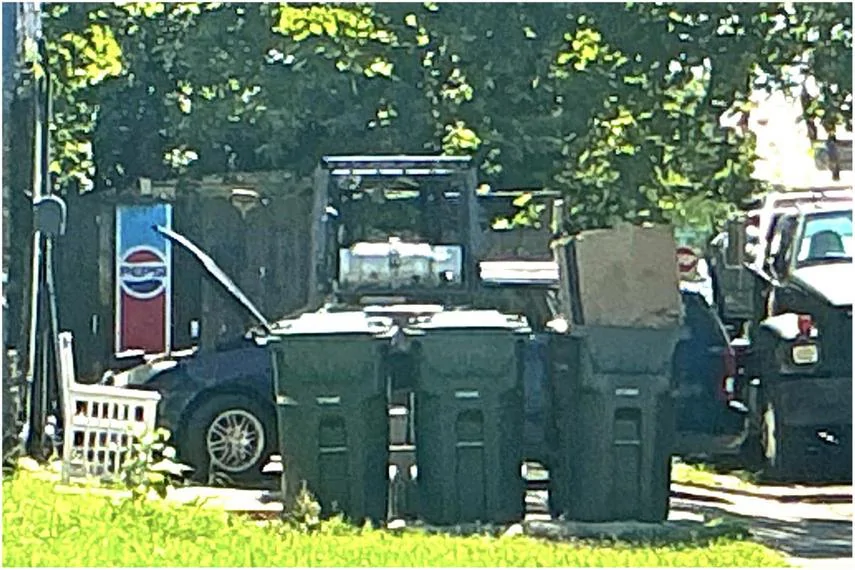
One in the shadow carries the largest load.

Three of the same garbage bins. They have the same job, right next to each other. One is more hidden, yet is doing the most for their job, and carrying the most.

To corroborate the idea that mental health concerns of Black girls are not a priority, Ayanna shared an image (Figure 12) that highlights the results of continuing to ignore these girls’ needs:

**FIGURE 12:**
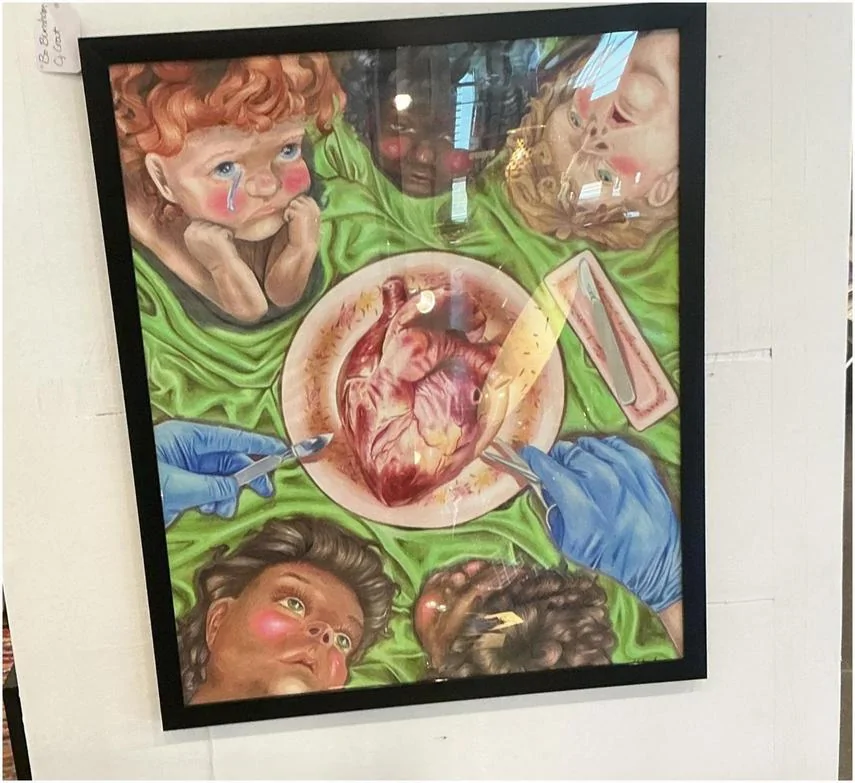
Until you’re dead.

Back in the 80s, people were prescribed cigarettes by doctors. It was seen as a relaxer, but later in the 2000s, the cigarettes were killing people and were finally seen as bad for you. I say this to show that many problems aren’t important till they kill many people or something big happens. Like the research problem, many kids have this problem [social media’s effect on the adultification of Black girls], but it isn’t seen as important because they are just kids. And it is easy to forget that kids can have problems and real feelings too.

The youth researchers continued to discuss different mental health concerns that Black girls may experience due to social media. They discussed with one another the possibility of being diagnosed with body dysmorphia due to the fact that Black girls are second-guessing their appearances when they post something online. They highlighted how peer approval and the perpetuation of adultification narratives can change their self-perception. Thus, one youth researcher, Ivory, reshared an image (Figure 12) that was interpreted as showing how social media can impact identity development. They went on to share more thoughts about the repercussions of children using social media and possible solutions:

It [the picture] can show people that introducing some little kids to the Internet takes away from their developing into their own true selves. What can we do about it? We can keep young kids off social media platforms until they have a confident idea of the person they’d like to be.

Furthermore, the youth researchers noted specific risk factors that can increase the likelihood of individuals having mental health concerns and unrealistic behavior changes when Black girls witness adultification narratives on social media:

…Especially if they’ve already been through like a traumatic incident like bullying or feeling like that, or just not being comfortable with who they are inside themselves. When they see those types of videos, I feel like they start to change themselves, so people around them will start to come toward them and be friendly.

## 5 Discussion

This study contributes to the limited YPAR literature focused on the experiences of Black girls in their communities during a time when social media has become an integral part of youth’s modern life. In this study, the authors used González et al.’s (2019) conceptualization of YPAR as a framework; their own initial research questions served as a guide, but the youth researchers’ co-created research question was at the center of the project. Additionally, a Black feminist, intersectional framework guided the study and the youth researchers’ research interests. This paper focused on their interest in the adultification and sexualization of Black girls via social media in a local city in the South. According to the youth researchers in this Southern city, social media has direct and indirect influences on their ability to be Black girls and has major implications for their quality of life. In this work, social media can be a tool to perpetuate gendered racism or a weapon against injustice and Western, white, heterosexual, middle-class ideology.

When analyzing their photos and group discussions, the youth researchers noted a shift in how they perceived what was considered innocent versus more adult-like behavior as they got older. This theme aligns with the findings from Goff et al. (2014), where Black children from the ages of 0–9 were perceived as innocent by adults, and this was similar to their peers from different races. On the contrary, the innocence of Black children who were between the ages of 10 and 13 was perceived as equivalent to that of peers of other races who were 14–17 years of age, and Black children who were 14–17 years of age had the same innocence perception as adults of other races (Goff et al., 2014). It is evident that Black children are perceived more prematurely than their peers, and it is noticeable, for Black girls, at a young age (Epstein et al., 2017). With more youth hyperfixated on sharing, creating, and communicating on social media (Abidin et al., 2025; De Leyn et al., 2022; Homayoun, 2018), it becomes easier for Black girls to experience and recognize adultification and understand the impact of being both Black and female on digital platforms when they are simply trying to be themselves.

Hence, while there are privacy procedures available to make sure youth are curating and engaging in “age-appropriate” content (Abidin et al., 2025), Black girls still may have their youthful content (e.g., participating in dance, outfit of the day trends, trying new hairstyles) marked as inappropriate due to gendered-racial bias toward Black girls and their behaviors, thus setting up an exchange of Black girls’ joy for broader acceptance based on dominant culture norms. A study from Haimson et al. (2021) supports this claim by revealing that social media content by Black creators is often removed from platforms for violating community guidelines, when in reality, they are being removed due to prejudice and systemic inequities. However, despite the online gendered and racial policing (Wade, 2019), Black girls deserve digital spaces (e.g., #BlackGirlMagic), where Black girls can build community with one another and retract negative, intersectional narratives about Black girls and women (Nashid, 2024; Rogers et al., 2021). For example, since systems of oppression, such as racism and sexism, will misinterpret the behaviors of Black girls, Halliday (2020) reshaped the narrative for twerking, a dance move that can be looked down upon or over-sexualized, and noted that it serves as a way for the author to express herself and experience joy.

Despite the increase in social media users and content creators, youth researchers noted a lack of Black women they can look up to on social media platforms. Research has shown that children want to choose mentors who are relevant to their lives and who they can compare themselves to Lockwood and Kunda (2000); Smooth and Richardson (2019). The youth researchers noted that while there are historical role models to admire (e.g., Martin Luther King, Jr., or Rosa Parks), they need more present-day role models who reshape narratives for Black girls. The youth researchers expressed that some Black girls may look up to prominent Black women on social media (e.g., female rappers) and use their behavior and careers as models for how Black girls and women should be represented. However, these individuals aren’t always viewed as respected role models for Black women in comparison to some historical figures, such as Michelle Obama (Leath and Mims, 2023), due to societal perceptions about these women, especially when many of these individuals’ (e.g., Cardi B, Meg the Stallion) leadership, civic, and community engagement are often not recognized on social media. However, regardless of the lack of recognition of “positive” behavior, notable Black women on social media should not be reduced to the gendered racist narrative and should be viewed holistically. Therefore, it may not be that Black girls lack role models but rather that they lack exposure to the work many Black women are currently doing. For example, Black women are historically underrepresented in discussions of academic success, such as pursuing STEM careers (Davis, 2020; Miles Nash and Peters, 2020), and similar to the girls in Troutman (2026), the youth researchers were yearning to learn more about other avenues for success beyond music or athletic careers, which are typically expected of them to explore (Women in Sport, 2025). The first step in doing so is to increase the visibility of the beautiful work and careers Black women have and are currently engaged in so Black girls can imagine unlimited possibilities for themselves. As an example, schools and community organizations can co-build spaces for Black girls where they can have conversations with Black women about their aspirations and dreams.

When youth use social media, they are often exposed to unrealistic expectations and mixed messaging from peers, parents, and other content creators (Homayoun, 2018; Kowalkowski and Smith, 2025). In return, youth may associate social success with the number of likes they receive on a post or video, or with the number of positive messages they receive (Homayoun, 2018). Youth researchers in this study noted that Black youth can be impressionable and often adjust their appearance and behavior to gain acceptance from others. Meaning, if Black girls are seeing individuals acting more mature (e.g., dressing a certain way, engaging in certain activities), they may view this as how to be successful in life and receive vicarious reinforcement (i.e., adopting the belief systems or behaviors of individuals of whom they consider desirable; Brown and Treviño, 2014). Homayoun supported this idea, noting that if youth compare their lives with others’ social success online, they may develop a fixed mindset that leads them to want to achieve the same success, which they can infuse into every part of their lives (2018).

Furthermore, when Black girls engage in what they see as social success, their behaviors may be adultified by their caregivers or close members of their community. For many Black families, there may be members who engage in respectability politics (i.e., engaging in behaviors that are socially acceptable by Western, white, heterosexual, middle-class norms as a survival strategy; Gould, 2009; Higginbotham, 1993), which can encourage socialization practices that lead to the vilification of Black girls and their actions (Leath and Mims, 2023). More specifically, family members may try to control the image of Black women to be more “respectable” rather than internalize stereotypical images of Black women (e.g., sapphire, jezebel, hood rat/ratchet chick; Hill Collins, 2000; Leath and Mims, 2023). The youth researchers confirmed the continuation of using respectability politics in this day and age, as they were aware of their family members’ ability to contribute to the adultification of Black girls, even if their intentions were rooted in protecting their children. The youth researchers desired a community where they could feel valued, heard, and occupy a space where they could be their authentic selves. Therefore, instead of expecting Black girls to change their behaviors to fit into dominant societal norms, family members should engage in behaviors that challenge negative depictions of Black girls (Leath and Mims, 2023), thus allowing them to be kids.

Messaging about the African Diaspora can include unconscious and conscious perceptions about skin-tone hierarchy and privileges, thus reinforcing colorism and the Eurocentric beauty as ideal. As the youth researchers noted, this type of messaging can start in the home or in one’s proximal community and be passed down to younger generations as preparation for bias; however, colorist ideologies can still harm Black girls, especially when social media can exacerbate the idea that lighter skin is celebrated, due to its proximity to whiteness, and darker skin is scrutinized. Many of the youth researchers discussed various opinions about colorism and colorist messaging and how these perceptions can change the behaviors of the Black girls. Crooks et al. (2025) reemphasized that colorism, which is often glorified on social media, has historically contributed to the adultification of Black girls by perpetuating the narrative around their sexual identity.

Dating back to slavery, women with darker skin were perceived as more masculine, angry, and stubborn (Blake et al., 2017), and are often hyper-sexualized and at-risk for criminalization (Sissoko et al., 2023), sexual violence (Opara et al., 2026), and internalization of negative self-concepts (Wallace et al., 2011) due to society’s beauty and dating standards. History is seemingly repeating itself, as the youth researchers shared honest observations about the negative online treatment of Black girls with darker skin tones. They constantly questioned why other individuals within or outside of the African Diaspora were able to “get away” with certain behaviors and natural physical appearance without judgment. Additionally, the youth researchers also noted that colorist messages on social media can unconsciously encourage Black girls to engage in behaviors that make them feel desired by others (Ward et al., 2023). However, despite acknowledging the impact of western beauty standards, the youth researchers were still able to recognize how fierce Black girls and women can be despite their skin tone, which aligns with Childs (2022) statement about how “our digital practice affords us the opportunity to self-define ourselves as beautiful, build community, and contest longstanding practices of exclusion in the beauty industry” (p. 14). Therefore, to begin the resistance toward the colorism that Black girls experience in their lives, virtually or in person, Rosario et al. (2021) suggested that Black girls can use positive affirmations related to their skin tone and recognize their strengths as individuals. Adult socializing agents, such as family members and school personnel, can encourage and share this positive narrative about Black girls as a way to start changing the narrative about Black girls’ appearance.

Digital spaces have the potential to be platforms that foster joy and positivity for Black individuals and engage in Black feminism (Childs, 2022; Taylor and Abidin, 2025). While social media can serve as a protective factor for Black girls (e.g., building digital kinship, increasing positive racial identity through media socialization; Rogers et al., 2021), the youth researchers in this study were in agreement about how certain platforms that perpetuate the narratives about Black girls being overly sexualized and deemed as less feminine (White et al., 2024) can increase mental health concerns (Anam and Harness, 2025; Homayoun, 2018). For instance, Black youth are more susceptible to experiencing stereotyping and racism on social media, which in turn increases their rates of depression and anxiety-related concerns (Anam and Harness, 2025; Hernandez et al., 2023). Because racial discrimination is a form of racial trauma (United States Department of Veterans Affairs, 2025), and Black youth are also exposed to more incidences related to discrimination, hate messaging, and bullying (Haimson et al., 2021; Tynes et al., 2019), it is not surprising that Black girls experience trauma-related symptoms, such as intrusive thoughts (Carter, 2007). Clark (2005) defined intrusive thoughts as recurring ideas that are unwanted, hard to control, and can be associated with negative emotions and poor self-concept. Similarly, the youth researchers expressed concerns about how social media can encourage Black girls with lower self-esteem to change who they are to get social approval, thus impeding their identity development, and explicitly noted the gendered racist stereotypes that they encounter when they typically are engaging in the same behavior or trends as their white counterparts. This finding aligns with Kowalkowski and Smith (2025) and Ward et al.’s (2023) findings that demonstrate how girls from racially minoritized backgrounds are more likely than white girls, to monitor their physical appearance, leading to mental health concerns related to their self-image, and grapple with expressing their authentic identity on social media when their group perception online is often reduced down sex objects or stereotypical character. More strategies, developed by Black girls themselves, are needed to provide realistic and relevant recommendations for continuing to utilize social media with joy and positivity while not engaging with whiteness.

Interestingly, while the girls in this study were able to identify the psychological impact of social media, the youth researchers expressed that their feelings were often neglected or misconstrued (Riddle and Sinclair, 2019). Leath and Mims reported that Black women tend to experience the dismissal of their emotions during their childhood and were often limited to show more acceptable emotions, if any, as a mechanism to avoid stereotypical labels and to be seen as strong (2023), which is a different parental experience from what white girls experience (Chaplin, 2015). Specifically, consistent with Leath and Mims (2023) findings, the girls in this study emphasized their frustration with caregivers and other community members’ lack of awareness of the stress and pressure they face, and how they often carry heavy, invisible burdens due to systems of oppression. Therefore, caregivers must acknowledge how social media platforms can be breeding grounds for psychological and physical concerns for Black girls due to gendered racism and then support their daughters’ social-emotional development by engaging in culturally responsive emotion socialization (i.e., teaching their children how to express and regulate their feelings and emotions based on their family’s cultural norms; Labella, 2018). On the other hand, the emotional experiences Black girls work through can also create opportunities to develop strengths-based skills such as self-advocacy, self-reflection, resistance, and critical judgment (Troutman, 2026). These skills can be used to help challenge and fight the systems they navigate daily.

### 5.1 Actionable change led by the youth

The ultimate goal of YPAR is for youth researchers to take action-based steps toward solutions in their community. When they analyzed their data, they reached a consensus on their next steps: in order to change outcomes in their community, they wanted kids to feel seen and heard. The youth researchers entered the research project not entirely sure what the main issues were in their community. Still, by the end of the project, they developed critical consciousness by consuming existing research, collecting and analyzing their own data, and understanding the implications of their answered research question for their community. The youth researchers acknowledged how social media can be a tool of systemic oppression and the various nuances that individuals within the African Diaspora experience.

The youth researchers worked as co-researchers, leading to several action-based steps to improve social media’s impact on the adultification of Black girls. First, similar to Kelly (2018), the girls decided to create social media platforms to raise awareness about the adultification of Black girls. Because of the platforms’ popularity, they created TikTok and Instagram accounts to share a Google Form survey and post videos and images about upcoming events and the outcomes of their outreach in the community. The Google Form survey focused on gaining more insight into the experiences of Black girls and Black women who were once Black girls, as it relates to adultification and sexualization in the community. Some survey responses were shared on social media to showcase the community’s views on the subject.

As part of their community outreach efforts, they attended a back-to-school block party to share their project with members of the community. During the event, they spoke with parents, students, and other community members about their research and explained why the topic is important. They also encouraged people to follow their social media platforms so they could continue learning about the project and its goals. In addition, they interviewed attendees to gather their perspectives and experiences on the topic, and they also took pictures and videos during the event to create content for their social media pages. Through this event, they engaged with the community, raised awareness, and shared their findings interactively.

In their most recent initiative, the youth researchers joined their local National Association for the Advancement of Colored People (NAACP) youth council to continue their advocacy work in the community. They were recognized for their efforts and celebrated for joining a collective of youth passionate about becoming change-makers. As part of this collective, the youth researchers hope to gain more opportunities to continue the conversation about the adultification of Black girls, learn more about civic engagement, and fight for needed change in their communities by using local resources.

### 5.2 Strengths

This study had several strengths. One strength was using a YPAR framework to shift what it means to conduct research with racially and ethnically minoritized youth. Instead of viewing the youth as participants, which is typically the case in traditional methodologies, or a population that needs to be studied, we saw them as youth researchers and partners for all stages of this research study (i.e., from developing a research question to disseminating the findings) (Tuck and Yang, 2014). Black youth are not something to be “studied”; they are humans with real life experiences and expertise who deserve agency in the research process when it involves providing recommendations in their communities. Furthermore, aligned with McCafferty et al. (2026) recommendations for ethical practice, the youth researchers in this study were compensated monetarily for their time and effort, thus acknowledging the expertise that they bring to the project and leveling the power dynamics. Another strength of this study is that the youth researchers were able to engage in actionable change that led to alignment with an organization with a shared message (Bertrand and Lozenski, 2023). Youth-led organizing collectives usually do not have the capacity to sustain action plans for long periods; thus, there is a need to mobilize with organizations of power to create and sustain space for youth decision-making, which the youth researchers in this study did by joining the NAACP’s youth council.

### 5.3 Limitations

As for limitations, during the data collection sessions, the youth researchers were supposed to take pictures with an iPad to help answer the research question they created as a homework assignment; however, they often forgot to take pictures due to their busy schedules and the lack of reminders from the adult researchers in between weekly sessions. Therefore, the photos in this study were taken only in a nearby area outside the community center, thereby restricting the range of photos. Furthermore, as in other photovoice studies (Salerno Valdez et al., 2019), the youth researchers were unable to photograph identifiable individuals to reduce the procedural load during data collection (e.g., blurring faces, obtaining photo consent) and to comply with the university’s IRB. Not providing this option to the youth researchers could have potentially limited their agency and creative freedom in engaging in Photovoice. Another potential limitation was using a criteria rubric for participation eligibility, which privileged certain students with academic abilities rather than being inclusive of all individuals who were part of the local school’s BSU. While the adult researchers included this rubric in case more than five individuals were interested, its intended use can be perceived as harmful and as gatekeeping. Finally, due to limited bidirectional communication between the youth and adult researchers after the summer and time constraints for the study, the youth participants were offered co-authorship on the manuscript; however, they were unable to commit to the time dedication required during the school year. During this time, it was also difficult to obtain pseudonyms from the youth participants after the study ended. The authors acknowledge that this lack of agency in the pseudonym-naming process should have been addressed in the YPAR sessions to ensure they could select names that represent them.

### 5.4 Future directions and implications

This study offers several important implications for practice. First, since Black girls spend a majority of their time at school, high schools can partner with community organizations to provide preventative initiatives and interventions on media literacy to Black girls, their caregivers, and members of their community to increase awareness about the adultification of Black girls on social media platforms (Anam and Harness, 2025). Initiatives and interventions, such as these, can also be the start of having open and honest conversations about their online experiences and the impact they have on their daily function (Love et al., 2021), and facilitate how Black girls can enjoy online spaces outside of respectability politics (Ohito and Nyachae, 2019). Therefore, partnering with schools, families, and community organizations is essential because, even though this paper focuses on the online adultification of Black girls, the impact of those negative messages and social interactions is present in the girls’ other aspects of life; thus, any supporting adult must be equipped with knowledge and strategies for how to effectively support Black girls. These interventions will also provide supporting adults with fundamental knowledge on how to combat this phenomenon from a systemic level, where schools and after-school programs can increase collaboration with Black girls (e.g., starting an advisory board) to co-develop solutions or school-wide policies to create more inclusive settings (e.g., creating culturally responsive anti-bullying programs that address digital behavior). Furthermore, schools can invest in culturally responsive or transformative social-emotional learning to give Black girls space to individually or collectively affirm their identity and counter narratives (Kubi et al., 2024). These intervention practices can be integrated in multi-tiered systems of supports, where at Tier 1 supports school-wide dissemination of media literacy and resistance strategies, Tier 2 focuses on skill-based group interventions with other Black girls (Scott and Collins, 2025) or sister circles (e.g., a safe space for collective healing, celebration of self with other Black girls; Dunmeyer et al., 2023), and Tier 3 focuses on providing tailored supports that meet the unique needs of individual Black girls.

For future research, scholars should consider the following suggestions. While other qualitative YPAR studies have worked with fewer youth researchers (Anderson, 2020), future studies should aim to recruit more researchers to capture more nuanced experiences of Black girls in this area. However, researchers can also use informational power to determine whether a smaller or larger sample size is needed, rather than relying on data saturation. Sampling guided by informational power can allow researchers to use smaller sample sizes if the individuals hold significant information relevant to the study’s aims (Malterud et al., 2021). Additionally, this study lasted approximately 4 months; therefore, future research should explore the impact of longitudinal studies, as there is very little information about the long-term benefits of participating in a YPAR project and how it affects one’s life over time (McCafferty et al., 2026). This study indicated the need for mental health and supportive dialogue for Black girls that also involves their close social network. Therefore, intervention-based research is needed to determine the effectiveness and social validity of interventions tailored to meet the needs of Black girls as it relates to enduring adultification and its ramifications. The authors also encourage future researchers to include their youth researchers at all stages of research, including the manuscript write-up, to ensure the accuracy of the findings and to prepare the youth researchers to disseminate the findings outside of the manuscript publication (Bordeaux et al., 2007). Furthermore, researchers should engage in refusal frameworks throughout all stages of inquiry by shifting the focus to systems of power rather than focusing strictly on minoritized individuals’ pain (Tuck and Yang, 2014). Literature centered around the investigation of the systems of oppression and opportunity is scarce, especially if Black girls are co-researchers instead of the subject (Javdani et al., 2011; Rose et al., 2024). Thus, there is a need for more scholarship that partners with Black girls to critically analyze the systems that they have to navigate.

## 6 Conclusion

This study contributes to the YPAR literature by situating Black female adolescents as experts on their own lived experience and providing them a space where their voices can be heard and uplifted. More specifically, this study adds to the literature about the mixed perception of social media platforms as it relates to Black girls and their experiences with gendered racism. While many studies focus on the adultification and sexualization of Black girls in physical communities, this study encouraged Black girls to be co-researchers and explore their experiences navigating social media platforms that perpetuate societal stereotypes or engagement in signs of resistance. The goal for YPAR projects is to facilitate actionable changes in the community. Therefore, the authors hope that adult socializing agents will prioritize listening to the Black girls in this study and collaborate with them in civic engagement that acknowledges, protects, and amplifies their voices. We also hope this work can inspire other Black girls to investigate all types of systems in their communities, including social media, and to see that they have the power to be the authors of their stories and promote change in their lives. The work is still not done; as social media continues to shape youth’s lives, researchers, educators, family members, and Black girls need to co-create digital spaces that affirm Black girls’ intersecting social identities, address inequities, highlight joy, and reflect their needs.

## Data Availability

The original contributions presented in this study are included in this article/supplementary material, further inquiries can be directed to the corresponding author.

## Footnotes

1 The authors are choosing to keep “white” lowercase to decenter whiteness in racial justice research.

2 In this study, due to the bidirectional agency between the research team and the students, the participants will be called youth collaborators to represent the expertise they embody.
